# Low-Dose Cyclophosphamide Associated With Hyponatremia and Hepatotoxicity

**DOI:** 10.7759/cureus.45375

**Published:** 2023-09-16

**Authors:** James R DeChiara, Eleanor M Birch, Hillary Harper

**Affiliations:** 1 Department of Emergency Medicine, Madigan Army Medical Center, Tacoma, USA

**Keywords:** case report, hepatotoxicity, acute liver injury, hyponatremia, drug toxicity, cyclophosphamide

## Abstract

Cyclophosphamide (CY) is an alkylating agent often used as a chemotherapeutic agent, with increasing use as an immunosuppressant. Cyclophosphamide has many established adverse effects, including hyponatremia and limited reports of hepatotoxicity, particularly in high-dose treatment. A case of simultaneous hyponatremia and acute liver injury associated with the initiation of cyclophosphamide two weeks prior is discussed here. A 73-year-old male with acquired hemophilia A/factor VIII deficiency presented to the emergency department (ED) with four days of hip pain and was found to have jaundice and confusion. Laboratory evaluation demonstrated hyponatremia and an acute liver injury associated with his recent cyclophosphamide use. With the discontinuation of the offending agent and sodium correction, he made a full recovery. Cyclophosphamide-induced hyponatremia is likely secondary to the nephrogenic syndrome of inappropriate antidiuresis (NSIAD) and is most often associated with high-dose regimens. While the mechanism of hepatotoxicity requires further study, it is likely dose-dependent and related to excess levels of 4-hydroxycyclophosphamide (HCY). The management of cyclophosphamide-induced water toxicity and hepatotoxicity is centered around the discontinuation of medication, the correction of electrolyte abnormalities, and supportive treatment.

## Introduction

Cyclophosphamide (CY) is a synthetic nitrogen mustard that was first developed for the treatment of malignancies. It is used both as a single agent and in combination for the treatment of lymphomas and solid tumors and for conditioning regimens [1]. Its use has been expanded to include nephrotic syndrome, rheumatoid arthritis, systemic lupus erythematosus (SLE), systemic vasculitis, and many additional rheumatologic and autoimmune diseases [2]. Medium- and high-dose regimens (up to 6000 mg/m^2^) were historically used in the treatment of malignancy, while low-dose courses of <10 mg/kg have been administered as an immunosuppressant [3]. Cyclophosphamide use is associated with significant toxicity. High-dose regimens generally produce more pronounced acute toxic effects, while the prolonged use of low-to-moderate-dose regimens can cause chronic toxicity [1]. Adverse effects include bone marrow suppression, amenorrhea, teratogenicity, bladder cancer, hemorrhagic cystitis, and hyponatremia [2]. Hyponatremia, more commonly induced by high-dose cyclophosphamide, can also occur with lower-dose regimens [4]. Additional uncommon adverse effects that require monitoring are hepatoxicity and cardiotoxicity [2]. We report a case of a 73-year-old patient who simultaneously developed acute life-threatening hyponatremia and hepatotoxicity two weeks after starting cyclophosphamide for acquired factor VIII deficiency.

## Case presentation

A 73-year-old male with a history of atrial fibrillation, right cerebellar stroke with residual aphasia, and acquired hemophilia A/factor VIII deficiency presented to the emergency department (ED) with four days of hip pain. He also reported nausea, intermittent lightheadedness, and fatigue since starting cyclophosphamide 100 mg daily for acquired hemophilia two weeks prior. Additional home medications included carvedilol, amlodipine, metformin, furosemide, spironolactone, and telmisartan. His physical examination was notable for jaundice and newfound confusion, in addition to his baseline aphasia. He had no abdominal tenderness or distention. Laboratory evaluation revealed a sodium of 115 compared with 134 approximately one month prior. His total bilirubin (Tb) was 4.50 and direct bilirubin (Db) 2.9 compared with 1.10 and 0.3, respectively, one month prior. Additionally, his alkaline phosphatase (ALP) and aspartate transaminase/alanine transaminase (AST/ALT) were increased to 326 and 190/366 from 69 and 14/17, respectively, also from one month prior. Synthetic liver function tests (LFTs) were normal. He had a nondetectable acetaminophen level and a nonreactive hepatitis panel. In the complete blood count, the eosinophil level was elevated to 9.2% of the differential. The computed tomography (CT) of the abdomen and pelvis revealed no abnormalities, and the right upper quadrant ultrasound showed normal parenchymal echogenicity and contour of the liver, appropriate directional flow, and normal biliary appearance.

In the ED, the patient was administered 100 mg of 3% hypertonic saline and N-acetylcysteine after consultation with the local poison center. He was placed on a free water restriction and admitted to the intensive care unit where cyclophosphamide was discontinued, and gastroenterology was consulted. Liver function tests (LFTs) uptrended over the next several days reaching a peak on day 5 with Tb of 10.1, Db of 8.3, ALP of 406, ALT of 312, AST of 161, and eosinophil levels of 16% of the white blood cell count (Table 1). The patient’s cyclophosphamide was permanently discontinued, and his laboratory tests improved with the complete normalization of all LFTs at the three-week follow-up.

**Table 1:** Pertinent laboratory data one month prior to admission (two weeks before starting cyclophosphamide), during the hospital course, and on follow-up three weeks later. Day 1 marks the patient’s ED visit and first day in the hospital. WBC, white blood cells; Na, sodium; BUN, blood urea nitrogen; sCr, serum creatinine; Tb, total bilirubin; Db, direct bilirubin; ALP, alkaline phosphatase; ALT, alanine transaminase; AST, aspartate transaminase; ED, emergency department

Laboratory Marker	Reference Values	Baseline (One Month Prior)	Day 1	Day 2	Day 3	Day 4	Day 5	Day 24
WBC (×10^3^/μL)	4.5-13.5	13.1	12.1	8.8	10.2	9.8	10	9.5
Eosinophil (%)	0-7.4	1.4	9.2	12.1	14.3	16.3	16	2.4
Na (mmol/L)	135-145	127	116	124	129	131	132	135
BUN (mg/dL)	6-23	11	9	7	8	11	10	12
sCr (mg/dL)	0.6-1.2	0.79	0.62	0.48	0.66	0.81	0.63	0.7
Tb (mg/dL)	0.15-1.2	1.1	4.5	6	6.9	9.7	10.1	2.1
Db (mg/dL)	0-0.3	0.3	2.9	4.3	5.4	7.2	8.3	0.7
ALP (U/L)	40-129	69	326	353	376	412	406	208
ALT (U/L)	0-41	14	366	359	346	312	312	31
AST (U/L)	0-50	17	190	198	176	152	161	21

## Discussion

**Figure 1:**
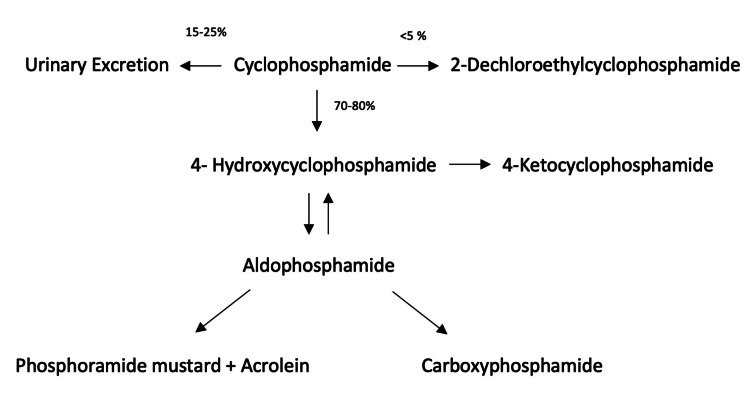
Metabolism of cyclophosphamide with the estimated breakdown quantification of administered dose.

Cyclophosphamide is a prodrug, requiring activation by the hepatic cytochrome P450 (CYP) system (Figure 1). Approximately 70%-80% of the cyclophosphamide dose gets activated to form 4-hydroxycyclophosphamide (HCY), mediated by CYP2B6 among other enzymes. HCY is in equilibrium with its tautomer aldocyclophosphamide, which circulates in the blood, can enter cells, and is degraded to phosphoramide mustard (PM) and acrolein [3,5]. Phosphoramide and acrolein are both cytotoxins, with acrolein having been identified as the causative agent in hemorrhagic cystitis in 1979 [6].

Water toxicity

Hyponatremia is a well-established complication of high-dose CY therapy, typically employed in transplantation and oncology. More recently, severe hyponatremia has been reported in patients taking low and moderate doses of CY for autoimmune diseases [7,8]. Bruining et al. summarized 12 cases of low-dose cyclophosphamide-induced hyponatremia, with most doses between 10 and 20 mg/kg but one as low as 50 mg and resultant serum sodium levels ranging from 106 to 116 [8]. More recently, Chen et al. reported a case of symptomatic hyponatremia in a patient treated for SLE with low-dose cyclophosphamide of 8 mg/kg and reviewed multiple cases of low-dose cyclophosphamide-induced hyponatremia [4]. Interestingly, the authors note that only five cases were associated with rheumatic diseases, most often SLE. None occurred during the treatment of acquired hemophilia [4].

Hyponatremia has been an increasingly reported side effect of low-dose CY use, is likely mediated through a direct tubular effect, and should be considered in all patients on cyclophosphamide. Strong evidence has recently emerged to suggest that the nephrogenic syndrome of inappropriate antidiuresis (NSIAD) is responsible for the hyponatremia associated with cyclophosphamide [9]. While authors first suggested that CY-induced hyponatremia may be due to the syndrome of inappropriate antidiuretic hormone secretion (SIADH) [10], subsequent cases arose in which arginine vasopressin (AVP) levels were unaltered during treatment with CY despite the development of hyponatremia, suggesting a direct effect of CY on the renal tubule [11,12]. Additionally, two cases of CY-induced hyponatremia occurred in patients with diabetes insipidus, adding further evidence to the hypothesis of a direct tubular effect [13,14]. In vitro experiments conducted by Kim et al. demonstrated that rat inner medullary collecting duct cells, cultured with 4-hydroperoxycyclophosphamide, produced higher levels of aquaporin-2 and vasopressin-2 receptor in the absence of AVP, further suggesting NSIAD as the mechanism of hyponatremia in cyclophosphamide use [15].

Hepatotoxicity

The first confirmed case of CY hepatotoxicity was in 1982 in a female treated for SLE who developed hepatitis during both initial treatment and a second challenge with cyclophosphamide. During the initial phase of treatment, she was treated with 1.5 mg/kg of CY and developed hepatitis six weeks into the treatment course. The medication was discontinued, and LFTs normalized. With increasing SLE symptoms six months later, the patient was restarted on CY 50 mg/day with close LFT monitoring and was found to have marked LFT elevation two weeks after restarting the medication [16]. Very limited additional instances of acute liver injury due to low-dose cyclophosphamide have been reported. Akay et al. described a male placed on 100 mg CY for the treatment of scleroderma who developed a hepatocellular liver injury pattern after six weeks of daily use, with the normalization of LFTs occurring 11 weeks after discontinuation [17]. Cleland and Pokorny reported on a female who developed an acute liver with hypereosinophilia eight weeks after starting 100 mg CY daily for SLE, with the normalization of LFTs occurring four weeks after discontinuation [18]. Snyder et al. presented a case of a female who developed hepatotoxicity at five weeks after starting 100 mg CY daily for granulomatosis with polyangiitis, with full recovery in seven weeks [19]. Finally, Subramaniam et al. reported a patient who developed acute hepatitis within 24 hours on two separate occasions, two weeks apart, after the administration of 200 mg CY. The patient’s ALT reached a level of 568 four days after the first dose, with improvement to 104 after two weeks, followed by an increase to 1253 one day later, after the second administration [20]. The case discussed here has many similar features to those previously reported with the rapid development of hepatotoxicity and the normalization of laboratory values weeks after the discontinuation of CY, which is attributed to the regenerative capacity of the liver, likely driven by cytokines, growth factors, and other signaling molecules [21]. Despite the similarities with the prior cases, to the author’s knowledge, this is the first reported case of combined low-dose cyclophosphamide-induced hyponatremia and hepatotoxicity.

Genetic factors affecting the intracellular detoxification of CY may contribute to individual susceptibility to liver injury. The intracellular detoxification of CY occurs predominantly through aldehyde dehydrogenase (ALDH) enzymes and glutathione S-transferases (GST). Polymorphisms resulting in alleles with decreased activity and the detoxification of 4-HCY may predispose patients on CY to a greater risk of liver toxicity. In a 2008 study, Ekhart et al. found that patients heterozygous for the ALDH3A1*2 and ALDH1A1*2 allele had greater rates of liver toxicity after undergoing high-dose cyclophosphamide chemotherapy regimens [22]. de Jonge et al. found that the greater exposure intensity of 4-HCY (measured by area under the plasma concentration-time curve) during the first course of treatment with CY was predictive of higher rates of veno-occlusive disease (VOD). There was no association between the area under the curve (AUC) of phosphoramide mustard and VOD, possibly because if PM (that is cytotoxic if found intracellularly) cannot enter cells, it has no cytotoxic properties [23]. A separate study found that patients with neoplasia treated with cyclophosphamide had significantly higher rates of liver dysfunction with total doses greater than 400 mg/m^2^ and in those with decreased urinary excretion of 3-hydroxypropyl mercapturic acid (3-HPMA), a major metabolite of acrolein, suggesting that CY-induced liver injury is dose-dependent and may be due to the impaired metabolism of both CY and acrolein [24]. McDonald et al. found a significant correlation of AUC of carboxyphosphamide and VOD in patients with hematologic malignancy undergoing allogenic transplant infused with 60 mg/kg/day of CY for two days followed by total body irradiation. However, the authors hypothesized that this is a marker of exposure to chemically reactive species formed from HCY, as it has been demonstrated that carboxyphosphamide is not a hepatotoxin; rather, it is a chemically stable CY metabolite between the formation of HCY and glutathione conjugates of acrolein and phosphoramide mustard [5]. While the exact mechanism of cyclophosphamide hepatotoxicity will require further research, it is likely that CY hepatotoxicity is dose-dependent and accentuated by the breakdown of CY metabolites, mainly 4-HCY.

## Conclusions

As CY is used more frequently for several rheumatologic diseases, additional attention must be paid to its adverse effects, even ones previously associated with high-dose regimens. This patient presented with a unique confluence of hyponatremia and hepatotoxicity secondary to his low-dose CY use for the treatment of acquired hemophilia. The mechanisms of these toxic effects are likely mediated through a direct tubular effect inducing hyponatremia and a dose-dependent exposure to the toxic metabolites of CY, mainly 4-hydroxycyclophosphamide, inducing hepatotoxicity. Clinicians must increasingly be aware of the adverse effects of medications in order to more effectively diagnose and treat their toxicities.
